# Idiocy

**Published:** 1895-01

**Authors:** J. W. Putnam

**Affiliations:** Professor of Nervous Diseases, University of Buffalo; 388 Franklin Street


### IDIOCY.

By J. W. PUTNAM, M. D.,
Professor of Nervous Diseases, University of Buffalo.

GENTLEMEN.—I have the opportunity of showing you, today, a number of idiots. Idiocy is a form of mental incapacity which must be differentiated from insanity. The latter is a disease developing in a brain previously healthy and well developed, while idiocy is a mental condition due to a poorly developed brain.

What are some of the causes of idiocy? In the first place, there is idiocy due to fetal causes. Secondly, we may have a perfectly developed fetus up to the time of parturition and then pressure upon the brain, due to the processes of labor or to the forceps, may cause either a meningeal hemorrhage or a thrombosis or a small submeningeal hemorrhage. Thirdly, we have, later in life, up to the age of four, during the period of development of the brain, eclampsic idiocy. By this is meant an idiocy coming on after the convulsions of teething and bowel troubles. Then we have an apoplectic idiocy which is developed before the age of seven. Again, there is microcephalic idiocy, which is due to a small skull, or one in which the fontanelles and sutures have closed too early to allow the proper expansion of the developing brain. In hydrocephalic idiocy, also, there is an undue pressure upon the brain. There may be trauma of the brain from falls and blows received during infancy and producing a hemorrhage or depression of the skull, etc. Then we have cretinism, which is a form of idiocy associated with goitre, which we rarely see in this country, but which is met with quite often in the Alps and the Himalayas. Again, there is an idiocy due to encephalitis or polio-encephalitis, which latter condition was assumed theoretically by Strümpel and afterward proved to exist in certain cases. Strümpel thought that this was the most common cause of idiocy, especially when associated with paralysis and spastic cerebral paralysis. Dr. Sachs, of New York, who has made an analysis of 140 cases of spastic cerebral paralysis, has determined that the most prominent causes are cerebral hemorrhage, cerebral embolism, and cerebral thrombosis. He does not agree with Professor Strümpel that this condition is analogous to the spastic paralysis of infants which is a polio-myelitis. In fact, he thinks that polio-encephalitis, which was put forward by

Strümpel as the most prominent cause of idiocy, is the least common cause.

As an illustration of this type of trouble, I show you a boy aged seven who is a twin. He was born at term, the breech presenting and forceps were applied to the after-coming head. He is of a far higher grade of idiocy than the child with athetosis. He is able to speak and to enjoy life. He looks at pictures, recognizes his friends, plays and has, apparently, no moral perversion such as is quite characteristic of the idiot. He has never had convulsions ; he has simply had this birth palsy.

There is a difference in the size of his hands, a difference in the length of the forearms and in the slope of the shoulders. The suspender will stay over the right shoulder, but not over the left. He does not rest on his heel, there being talipes equinus. The development of the bones and muscles of both limbs on the left side, and of the left side of the head and face, is less than on the right side. The palate is highly arched, the teeth are fairly good, the temporal regions of both sides are depressed so that the skull is narrow in the bi-temporal diameter. You will notice a flexion of the wrist and a certain degree of spastic condition. Infantile spastic cerebral paralysis may be monoplegic, hemiplegic or diplegic.

In a case of this degree of idiocy, what is to be done ? Ordinarily, a child of this grade of intellect cannot get along at the common school with the usual methods of teaching. He can be educated, however, by going to a special school, where particular attention and study is given to teaching inferior intellects. There is such a school at Syracuse. This patient is a little irritable, but he is healthy and he promises a good deal. He can be taught some trade beside other accomplishments.

The third case is one of microcephalus in a child of four. The patient is unable to stand, unable to coördinate its movements and is able to say only half a dozen words. The cause of the trouble in this child is the early closing of the sutures and fontanelles of the skull. The relief of this form of idiocy has been unsuccessful until the last three years. Within that time, surgeons have devised an operation which has proved successful in a number of cases. The operation consists in channeling with a chisel along each side of the sagittal suture and running channels also in other directions, according to the shape and the depressions of the skull. Sometimes the additional grooves are made from the anterior fontanelle downward and from the posterior fontanelle backward. This operation gives the brain a chance to grow if it will develop. Certain cases published by Lannalongue, and one or two by W. W. Keen, and several by other operators, including our own surgeons, Park and Mynter, have shown good results. I think, therefore, that in a case of idiocy due to microcephalus we are justified, rather than to allow the child to spend its life in hopeless helplessness, to advise such an operation. There is, apparently, no danger in the craniotomy. It is simply a bone operation, and the brain itself receives no exploration and no injury. The risk that is run is slight compared with the chances for improvement.

There is one form of idiocy which I have not yet mentioned, the idiocy of deprivation ; that is the form which is caused by the absence of one or more of the senses. Such a case was that of the famous Laura Bridgeman, who had none of her senses except touch, yet, by the development of that sense at the expense of a great deal of labor, the most that could possibly have been made of her intellect was effected. But a patient who is born deaf, dumb or blind has a latent cause of idiocy, and if not properly trained by masters who understand the development of the mind under this serious handicap, then the child is more than likely to develop idiocy due to the deprivation.

388 Franklin Street.

